# Tuberculosis in older adults: challenges and best practices in the Western Pacific Region

**DOI:** 10.1016/j.lanwpc.2023.100770

**Published:** 2023-04-20

**Authors:** Alvin Kuo Jing Teo, Fukushi Morishita, Tauhid Islam, Kerri Viney, Catherine W.M. Ong, Seiya Kato, HeeJin Kim, Yuhong Liu, Kyung Hyun Oh, Takashi Yoshiyama, Akihiro Ohkado, Kalpeshsinh Rahevar, Lisa Kawatsu, Manami Yanagawa, Kiesha Prem, Siyan Yi, Huong Thi Giang Tran, Ben J. Marais

**Affiliations:** a Saw Swee Hock School of Public Health, National University of Singapore and National University Health System, Singapore, Singapore; b Faculty of Medicine and Health, University of Sydney, Sydney, NSW, Australia; c The University of Sydney Institute for Infectious Diseases (Sydney ID) and the Centre of Research Excellence in Tuberculosis (TB-CRE), Sydney, NSW, Australia; d World Health Organization, Regional Office for the Western Pacific, Manila, Philippines; e World Health Organization, Global Tuberculosis Programme, Geneva, Switzerland; f Infectious Diseases Translational Research Programme, Department of Medicine, National University of Singapore, Singapore, Singapore; g Division of Infectious Diseases, Department of Medicine, National University Hospital, Singapore, Singapore; h Institute of Health Innovation and Technology (iHealthtech), National University of Singapore, Singapore; i Research Institute of Tuberculosis, Japan Anti-Tuberculosis Association, Tokyo, Japan; j Korean National Tuberculosis Association, Seoul, Republic of Korea; k Beijing Chest Hospital, Capital Medical University, Beijing, China; l Department of Infectious Disease Epidemiology, London School of Hygiene and Tropical Medicine, London, UK; m KHANA Center for Population Health Research, Phnom Penh, Cambodia; n Center for Global Health Research, Public Health Program, Touro University California, Vallejo, CA, USA

**Keywords:** Tuberculosis, Aged, Policy

## Abstract

The Western Pacific has one of the fastest-growing older adult populations globally, and tuberculosis (TB) remains one of the foremost infectious causes of disease and death in the region. Older adults are at higher risk of TB due to immunosenescence, comorbidities, and increased institutionalisation. Atypical symptoms and reduced access to health services may delay care-seeking and TB diagnosis, while co-morbidity and increased risk of adverse drug reactions complicate TB treatment. Post-TB sequelae and socioeconomic challenges may decrease the quality of life after TB treatment completion. Despite their high disease burden and special challenges, there is a lack of regionally coordinated policies and guidelines to manage TB among older adults. Routine TB screening at aged-care facilities, age-friendly infrastructure and services, awareness of atypical TB features, integration of TB and non-communicable diseases services, and person-centred approaches to treatment support could improve TB management among older adults. Addressing these challenges and adopting the best practices identified should inform policy formulation and implementation.

**Funding:**

This project was funded by 1) the 10.13039/100004423World Health Organization Regional Office for the Western Pacific, with financial contributions from the Government of the Republic of Korea through the Korean Disease Control and Prevention Agency and the Government of Japan through the 10.13039/501100003478Ministry of Health, Labour and Welfare, and 2) 10.13039/501100001352NUS Start-up Grant. The funders had no role in the paper design, collection, analysis, and interpretation of data and in writing of the paper.

## Background

The Western Pacific Region, home to 1.9 billion people in 37 countries and areas, has one of the largest and fastest-growing populations of older adults globally. The average life expectancy of people in the region was 77.7 years in 2019, 4 years above the global estimate (73.3 years).^1^^,^^2^ The increased TB risk of this ageing population poses a major public health challenge. While TB deaths have gradually declined across all age groups since the 1990s, TB remains one of the leading infectious causes of death worldwide, with increasing age recognised as a major risk factor for TB-related death.^3^^,^^4^ In 2021, the estimated incidence in the Western Pacific Region was the highest among persons aged ≥65 years (men—255,000 and women—116,000), accounting for 19.5% of the total TB incidence.^5^ Between 2020 and 2021, people with TB aged ≥65 years made up a large proportion of the notified TB cases in countries and areas such as Japan (69%), the Republic of Korea (51%), and Hong Kong Special Administrative Region, China (hereinafter Hong Kong SAR) (45%).^5^

Failure to control and manage TB among older adults places them at risk and could also perpetuate transmission to other age groups. Given the absence of regional guidance on how best to deal with this challenge, a careful assessment of the situation is required to assist countries in meeting ambitious End TB Strategy targets.^6^ In this article, we describe the risk factors for TB disease and transmission and the clinical disease manifestations of TB in older adults, with a focus on the Western Pacific Region. We also highlight key challenges, relevant strategies, and associated research priorities related to TB transmission, detection, diagnosis, treatment, and post-TB health (See on-line Supplementary Material for a detailed description of the literature search methods and limitations).

### Contextual heterogeneity

The Western Pacific Region includes a diverse range of countries and areas with different population structures, cultural beliefs and social norms, economic resources, and healthcare systems. This heterogeneity reflects differences in TB transmission risk, as well as risk factors for disease development, which contributes to variable disease burden observed among older adults in different countries and states (Table 1).

**Table 1:** Epidemiological profiles and variable TB incidence among older adults in countries and areas in the Western Pacific Region (2021).

Countries and areas	Country income classification^7^^,^^f^	UHC indices of service coverage 2019^8^^,^^d^	TB incidence rate per 100,000 population per year^5^^,^^e^, ^f^	Epidemiological classification^9^ for TB based on incidence rate^5^^,^^e^, ^f^	Estimated TB case load^5^^,^^e^, ^f^	Estimated TB incidence in older adults^5^^,^^e^, ^f^	% of incident TB cases in older adults^5^^,^^f^	BCG coverage^10^^,^^c^
Western Pacific Region		80^11^	98		1,900,000	371,000	19.5%	89.0%
Philippines^a^	Lower-middle	55	650	Severely endemic	741,000	78,000	10.5%	47.0%
Marshall Islands	Upper-middle	N/A	483	Highly endemic	200	10	5.0%	83.0%
Mongolia^a^	Lower-middle	63	428	Highly endemic	14,000	1080	7.7%	99.4%
Papua New Guinea^a^	Lower-middle	33	424	Highly endemic	42,000	920	2.2%	42.0%
Kiribati	Lower-middle	51	424	Highly endemic	550	35	6.4%	96.0%
Tuvalu	Upper-middle	N/A	296	Endemic	33	6	18.2%	100.0%
Cambodia^b^	Upper-middle	61	288	Endemic	48,000	8900	18.5%	92.0%
Nauru	High	N/A	193	Endemic	24	2	8.3%	100.0%
Viet Nam^a^	Lower-middle	70	173	Endemic	169,000	41,000	24.3%	87.9%
Lao People's Democratic Republic	Lower-middle	50	143	Endemic	11,000	2010	18.3%	81.4%
Malaysia	Upper-middle	76	97	Upper moderate	33,000	5400	16.4%	99.0%
Northern Mariana Islands	High	N/A	81	Upper moderate	40	7	17.5%	13.0% (2010)
Micronesia (Federated States of)	Lower-middle	48	80	Upper moderate	90	0	0.0%	59.2%
Fiji	Upper-middle	61	66	Upper moderate	610	49	8.0%	99.6% (2020)
Solomon Islands	Lower-middle	50	65	Upper moderate	460	40	8.7%	83.5%
Brunei Darussalam	High	77	61	Upper moderate	270	58	21.5%	99.9%
China, Macao SAR	High	N/A	57	Upper moderate	390	143	36.7%	99.7%
China, Hong Kong SAR	High	N/A	57	Upper moderate	4300	1920	44.7%	95.0%
China^a^	Upper-middle	82	55	Upper moderate	780,000	210,000	26.9%	99.7%
Palau	Upper-middle	N/A	51	Upper moderate	9	0	0.0%	N/A
Niue	N/A	N/A	48	Lower moderate	0	0	0.0%	88.0%
Singapore	High	86	48	Lower moderate	2800	720	25.7%	98.0% (2018)
Republic of Korea	High	87	44	Lower moderate	23,000	11,700	50.9%	98.0% (2019)
Guam	High	N/A	39	Lower moderate	67	10	14.9%	N/A
Vanuatu	Lower-middle	52	34	Lower moderate	110	9	8.2%	76.0%
Tokelau	N/A	N/A	19	Upper moderate	0	0	0.0%	100.0%
French Polynesia	High	N/A	13	Lower moderate	39	8	20.5%	96.0% (2019)
Cook Islands	N/A	N/A	13	Lower moderate	2	0	0.0%	100.0%
Japan	High	85	11	Lower moderate	13,000	9200	70.8%	95.0% (2020)
New Caledonia	High	N/A	10	Lower moderate	29	14	48.3%	95.0% (2018)
Tonga	Upper-middle	56	7.6	Low incidence	8	2	25.0%	100.0%
New Zealand	High	86	6.8	Low incidence	350	54	15.4%	9.9%
Samoa	Lower-middle	53	6.8	Low incidence	15	3	20.0%	92.0%
Australia	High	87	6.5	Low incidence	1700	290	17.1%	N/A
American Samoa	Upper-middle	N/A	4.1	Low incidence	2	1	50.0%	91.0% (1998)
Wallis and Futuna Islands	N/A	N/A	1.9	Low incidence	0	0	0.0%	97.0% (2016)
Pitcairn Islands	N/A	N/A	N/A	N/A	N/A	N/A	N/A	N/A

BCG; Bacillus Calmette–Guérin, N/A; data not available, SAR; special administration region, TB; tuberculosis, UHC; Universal health coverage.

a WHO high TB burden country.

b Recently removed from the WHO high TB burden list and was included on a global TB watchlist.

c Latest data (2021) is presented unless otherwise stated. If official, administrative, and WHO/UNICEF estimates are provided for the same year, official data is presented.

d Average coverage of essential services based on tracer interventions that include reproductive, maternal, newborn and child health, infectious diseases, non-communicable diseases and service capacity and access, among the general and the most disadvantaged population. The indicator is an index reported on a unitless scale of 0–100, with 0 being the worst, and 100 the best.

e These estimates (including those used for grouping) have uncertainty ranges but only best estimates are provided in the table and were used for calculating % of incidence TB cases (age ≥65 years).

f All data for 2021 (as stated in heading, unless otherwise specified).

In settings with a lower TB burden and limited community transmissions, such as Hong Kong SAR, Japan, and Singapore, TB incidence among older adults is mainly driven by increased rates of reactivation disease.^12^, ^13^, ^14^ In high-burden settings such as Cambodia and Viet Nam with evidence of ongoing community transmission, older adults are at risk of developing TB disease through both reactivations of past TB infection and disease progression following recent primary or re-infection.^15^^,^^16^ China demonstrates a country in transition where enhanced TB management has reduced TB transmission within communities, leading to a relative increase in reactivation disease among older people in the coming decades.^17^ Given various TB disease burdens in the Western Pacific Region,^18^ both reactivation pathway and exogenous infection/re-infection pathway should be considered, especially in households and congregate settings such as aged care facilities and hospitals.^13^^,^^16^^,^^19^^,^^20^ Institutional transmission among residents and staff within and between aged-care and health facilities has been reported,^13^^,^^20^^,^^21^ posing a major infection control challenge, particularly if disease detection is delayed.

Increasing immunosenescence in older individuals, a process of age-associated immune dysfunction, increases the risk of TB reactivation.^19^^,^^22^^,^^23^ In addition, the presence of other comorbidities that increase with age, such as diabetes, could experience ≥1.5-folds increased risk of developing TB.^24^, ^25^, ^26^ The prevalence and severity of chronic respiratory disease, including chronic obstructive pulmonary disease (COPD) and bronchiectasis, increase as a person ages.^27^ The relationship between chronic respiratory disease and TB, both as a cause and a consequence, is more significant in high TB incidence settings.^28^ Another condition prevalent among older adults is undernutrition,^29^ especially among those living in aged care facilities.^30^ Undernutrition leads to impaired immune function, thereby increasing the risk of TB reactivation and the risk that a new infection will rapidly progress to TB disease.^31^^,^^32^ The detrimental synergistic effect of ageing, diabetes, and undernutrition on the immune system elevates the vulnerability of older adults to develop TB, including from drug-resistant strains.^24^^,^^33^ With improved health care, people living with HIV are also ageing and therefore are at risk of similar age-related comorbidities.^34^

## Clinical disease manifestations

Typical pulmonary TB symptoms such as prolonged cough, haemoptysis, night sweats, chills, fatigue, and loss of appetite are less prominent in older adults with TB,^35^^,^^36^ while those with extrapulmonary TB usually experience symptoms localised to the site of disease. Disseminated TB, which more frequently occurs in immunocompromised individuals or people with immunosenescence, is associated with non-specific symptoms such as unexplained fever and weight loss.^35^ The presentation of TB-associated symptoms may also be confounded by concurrent comorbidities, making TB more challenging to diagnose in older adults. Radiological features also differ between younger and older adults,^37^ with older adults less likely to have lung nodules or consolidation on computed tomography (CT)^38^ or typical apical lung cavities on the chest radiograph.^39^, ^40^, ^41^ The presence of lung nodules and soft tissue masses is also more likely to indicate malignancies in older adults, which may co-exist with TB disease.

## Key challenges in addressing TB among older adults

**Fig. 1:**
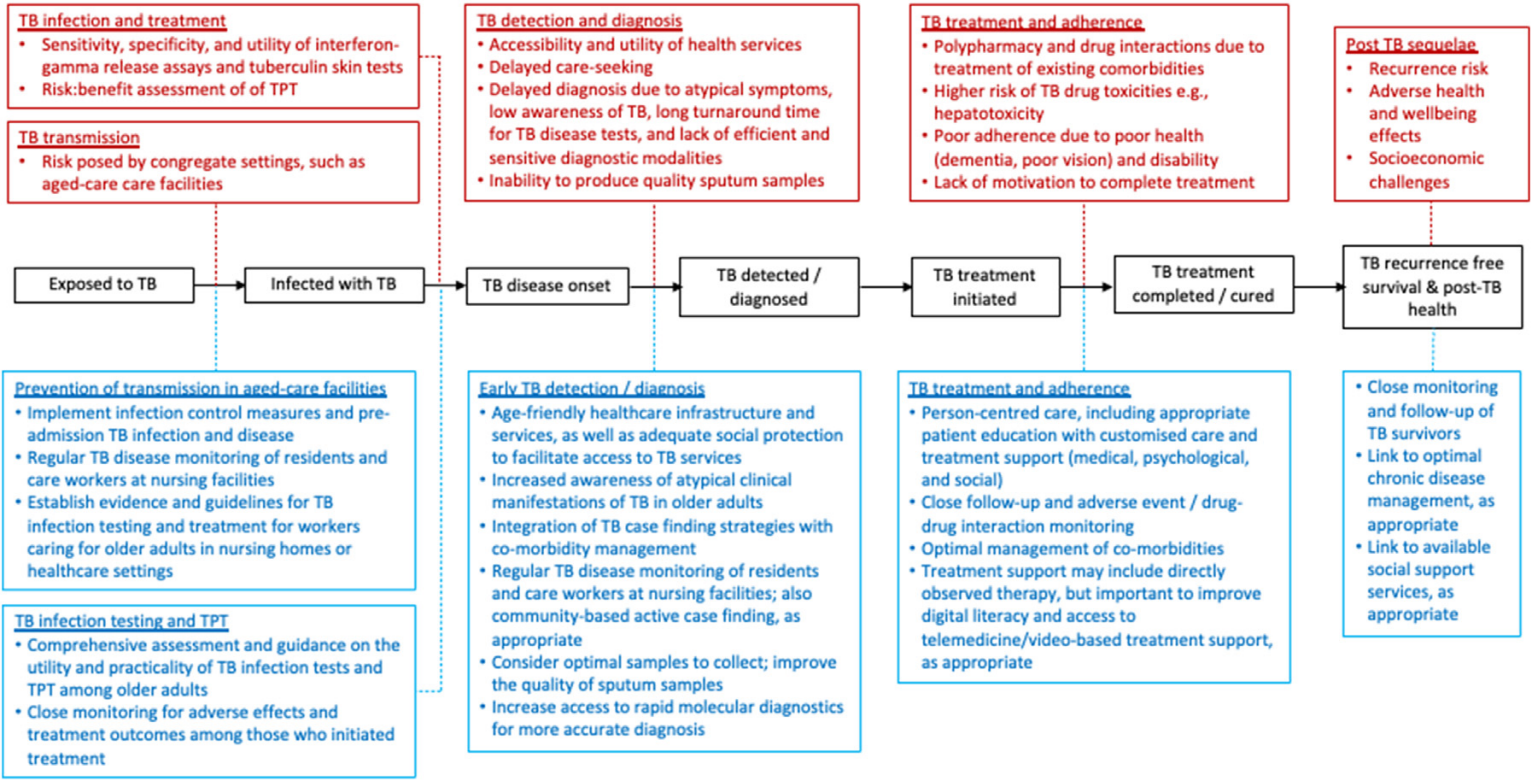
Key challenges faced by older adults along the TB care pathway and the corresponding best practices identified to overcome these barriers. Red boxes represent key challenges, and blue boxes reflect best practices to address them. TB; tuberculosis, TPT; TB preventive treatment.

Fig. 1 and Supplementary Table S1 provide an overview of key challenges along the TB care cascade with a brief description of the best practices identified to overcome them.

### TB infection management

Aged-care facilities where TB infection is common in older people represent congregate settings with a high risk of TB transmission. In general, the WHO recommends the traditional tuberculin skin test (TST) and newer interferon-gamma-releasing assays (IGRAs) to test for TB infection. Nonetheless, age-related immunosenescence may reduce overall sensitivity,^42^, ^43^, ^44^, ^45^ although newer generation IGRAs have reported better performance in detecting TB infection among older adults.^46^ The utility of TST for TB infection testing might also be affected by Bacillus Calmette–Guérin (BCG) vaccination status (BCG coverage by countries and areas is provided in Table 1),^47^ and nutritional status.^48^ The systematic screening of older adults for TB infection using TST or IGRA is not currently recommended by the WHO.

A recent study among aged-care residents favoured IGRA over TST in predicting future TB disease.^38^^,^^49^ In practice, the preferred test will depend on local availability, accessibility, and cost,^50^^,^^51^ especially in low-income settings. The adoption of TB infection screening strategies among older adults in the national policies also relies on the availability of evidence, including risk-benefit assessments, which are currently lacking.

Apart from the residents, the caretakers and staff at these facilities are also at risk of TB infection and disease, contributing to TB transmission.^52^, ^53^, ^54^, ^55^, ^56^ In China, TB preventive treatment (TPT) among older adults was projected to be the single most impactful intervention to reduce TB incidence and mortality.^17^ The WHO now conditionally recommends expanded TPT for children ≥5 and adults (in addition to children <5 years) in household contact with someone who has bacteriologically confirmed pulmonary TB who does not have active TB disease. However, the greater risk of drug-related toxicity in older adults is acknowledged with uncertain risk-benefit ratios.^57^ TPT has important short- and long-term benefits in preventing TB disease among those with TB infection or re-infection.^57^ TB infection testing and TPT were also recommended for other household contacts of people with bacteriologically confirmed TB and other at-risk populations, such as those receiving tumour necrosis factor inhibitors, dialysis, and organ/haematological transplants. Consideration should also be given to incarcerated people, health workers, homeless people, and people who use drugs.^57^ In general, however, systematic treatment of older adults for TB infection is not recommended by WHO at present. The adverse effects of the TPT regimen, such as the hepatic adverse effects of isoniazid, the most commonly used TPT drug, in older adults, remain a concern.^58^^,^^59^ Moreover, in a survey among national TB programs of high-burden countries (including lower-middle-income economies in the region: Philippines, Papua New Guinea, Cambodia, and Viet Nam), the lack of time and funding for procurement and implementation of the safer and shorter TPT regimen was reported as barriers to their inclusion in the national policies.^60^

### TB diagnosis and detection

#### Delayed TB care-seeking

Older adults may face significant challenges in accessing TB services, which can be attributed to various socio-economic and health system challenges. Challenges include a lack of comprehensive support systems to help manage the complex health issues that disproportionately affect this population, as well as physical and financial barriers that impede healthcare access.^61^, ^62^, ^63^, ^64^, ^65^, ^66^ In situations where social security and protection schemes are inadequate, older adults with limited financial means or independence may be reluctant to impose on others to initiate the care-seeking process.^66^ This could potentially result in more advanced disease at the time of diagnosis, making treatment more difficult and expensive, and incurring a greater cost burden on the person and household affected by TB. For instance, in China, the median time from symptom onset to TB disease diagnosis was >90 days among older adults,^67^ and this population has 4 times the odds of experiencing catastrophic costs due to TB compared to younger adults.^68^

A systematic review that assessed healthcare access among older females living in rural settings reported long waiting times (reported by studies in Nigeria, India, the United States of America, and China), limited medical resources (reported by studies in Ghana and South Africa), and poor attitude of healthcare staff towards older adults (reported by studies in South Africa, India, and China), as barriers to health service utilisation.^69^ In Cambodia, private healthcare-seeking was common upon falling ill with TB, as it was perceived to provide better care and higher quality medicines.^70^ However, access to TB diagnostics in the Cambodian private sector was reported to be limited.

Older adults are more likely to dismiss TB symptoms due to concurrent medical conditions, such as existing cough due to smoking-induced chronic lung disease, misconceptions of TB disease risk, and fear of discrimination, leading to delayed care-seeking and TB diagnosis.^62^^,^^64^^,^^71^ Inadequate TB awareness and knowledge were also documented among older adults who reported lower TB knowledge, attitude, and practice scores in China.^72^ However, few studies explored care-seeking behaviours and other predictors of delayed TB diagnosis among older adults in the Western Pacific Region. In general, TB care-seeking behaviour is influenced by local cultural practices, beliefs, and social norms,^73^ as well as universal challenges related to healthcare cost, accessibility and perceived levels of care.

#### Delayed diagnosis

On presentation to health facilities, TB is often misdiagnosed due to atypical presentation in older adults.^55^^,^^74^^,^^75^ TB diagnosis in older adults is further complicated by challenges related to advanced age, such as cognitive impairment, hearing loss, and communication difficulties.^71^ Non-specific TB symptoms and low awareness among health practitioners,^76^ especially in lower TB incidence settings, also contribute to delayed diagnosis. Between 2012 and 2016, data from the national health insurance system in the Republic of Korea showed that 7186 people diagnosed with TB were not recognised on initial hospitalisation; the majority (64%) of those with initial missed diagnosis were aged >60 years.^77^ Clinicians’ lack of responsiveness to health concerns expressed by older adults has also been perceived as a barrier to health care delivery.^78^

A critical factor in determining the validity of any sputum-based test is the quality of the sputum specimen provided, and older adults may struggle to produce an adequate expectorated sputum sample.^15^ An assessment of sputum smear results in four countries (including Mongolia) reported higher proportions of low-grade positive smears reported in populations at the extremes of ages.^79^ Samples with low bacillary load reduce the sensitivity of microscopic examination, resulting in missed cases of TB.^79^^,^^80^ Delays in TB diagnosis and treatment initiation, as well as inappropriate isolation facilitates nosocomial TB transmission.^31^ There is a wide spectrum of TB disease risk and presentation across finer age groups,^81^ with the extent of delay in care-seeking, diagnosis and treatment and the outcomes of TB treatment generally worse in the older age groups.^41^ This may have implications for aged-based surveillance and tailored treatment.

### TB treatment and adherence

#### Drug–drug interactions due to the treatment of existing comorbidities

Polypharmacy, often defined as the simultaneous use of ≥5 medications, is common among older adults. A recent systematic review reported a pooled prevalence of 37% (36% among studies conducted in Asia).^82^ A separate systematic review conducted among residents of aged-care facilities reported the prevalence of polypharmacy ranging from 38% to 91%.^83^ The pooled prevalence of polypharmacy among older adults in China was estimated to be 48%, with three-quarters occurring in in-patient settings.^84^

For older adults with TB, the co-administration of TB treatment with drugs used to manage co-morbid conditions frequently lead to drug–drug interactions that may adversely affect clinical outcomes. For instance, rifampicin could reduce the therapeutic levels of some drugs, such as the antiarrhythmic drug amiodarone, the anticoagulant warfarin and other novel oral anticoagulants, and anti-diabetic drug rosiglitazone.^85^ Isoniazid may cause increased hepatotoxicity if used with paracetamol, alcohol, or valproic acid.^85^ The use of common over-the-counter medications, such as antacids, could also reduce the concentrations of isoniazid and rifampicin.^85^

#### Higher risk of TB drug toxicities

In general, older adults are at higher risk of adverse drug events than younger people. Increasing age is a risk factor for hepatotoxicity associated with isoniazid and rifampicin.^31^ The risk of pyrazinamide-related adverse events also increases with age, with liver toxicity and gastrointestinal intolerance being the most commonly reported side effects.^86^^,^^87^ For older adults who are unlikely to tolerate pyrazinamide, a treatment regimen of 9 months without pyrazinamide (2 months of ethambutol, rifampicin, and isoniazid; and 7 months of rifampicin and isoniazid) has been used.^88^ However, studies in Japan reported that pyrazinamide-containing TB regimens did not lead to significantly higher treatment discontinuation rates, liver toxicity, or death than regimens without pyrazinamide among older adults.^89^, ^90^, ^91^ Nevertheless, even in the absence of pyrazinamide, the frequency of treatment-related adverse events was generally higher in older adults with TB.^92^ Ethambutol, for instance, has been reported to be the most common drug responsible for major adverse events (e.g., dermatologic, gastrointestinal, arthralgia, liver injuries, and visual changes) among older adults in the Republic of Korea.^93^ Its potential effect on visual acuity should be considered in the presence of other age-related eye problems such as retinopathy and cataracts.^31^ Among second-line TB drugs,^94^ fluoroquinolone use among older adults has been associated with a higher risk of hepatoxicity, tendinopathy, neuropsychiatric reactions, and QT prolongation.^95^ There is a lack of safety and efficacy data on bedaquiline use in older adults,^96^ and the use of linezolid (the third group A drug) has been associated with a higher risk of serious adverse events, particularly if used for longer periods.^94^ In general, there is a lack of safety data on linezolid among older adults.^97^^,^^98^ Most group B and C second-line TB drugs^94^ have also been reported to be either less tolerable among older adults or to lack age-specific data.^74^^,^^99^

Traditional, complementary, and herbal medicines are ubiquitously used in this region.^100^ A meta-analysis of randomised controlled trials in 2020 reported that combination therapy of various traditional Chinese medicines and standard TB treatment regimens had higher pulmonary lesion absorption and cavity closure rates than the control group (standard anti-TB regimen).^101^ However, the evaluated studies lacked rigour and were poorly standardised. The use of concurrent herbal medicines is generally discouraged, given their association with drug-induced liver injury.^100^ Ironically, its use as a ‘liver protector’ is pervasive among people on TB treatment, especially in China, despite a lack of evidence to support the practice.^102^ Further systematic evaluation is required.

#### Poor adherence due to health issues and inadequate support

TB treatment adherence is vital in ensuring a favourable outcome. However, loss to follow-up^103^ and treatment adherence are often an issue among older adults due to poor health understanding and general disability, as well as more frequent adverse events and drug–drug interactions.^31^^,^^104^, ^105^, ^106^ Cognitive impairment, mostly dementia, has also been associated with poor adherence to TB treatment.^107^ All these factors justify additional and tailored support to facilitate safe treatment completion.^31^

### Post-TB health and rehabilitation

While TB is curable, TB survivors often experience long-term health sequelae, including the risk of future TB recurrence and ongoing socioeconomic challenges even after being cured. Studies conducted among older adults reported decreased lung functions and higher severity of COPD among TB survivors in the Republic of Korea.^108^^,^^109^ The risk of TB-related chronic lung disease is highest in high TB burden countries and among cigarette smokers.^28^ TB survivors also experience a significantly increased risk of death compared to the general population post-treatment, with most deaths attributed to cardiovascular disease.^110^, ^111^, ^112^, ^113^ TB and disabilities associated with it may have multiple negative impacts on a survivor's quality of life^114^, ^115^, ^116^ and mental health, compounded by TB-associated stigma.^117^ Apart from direct health impacts, the socioeconomic implications of TB may also endure post-treatment completion. TB-affected households report ongoing income loss and lack of employment beyond TB treatment completion, which also affects older adults in these households.^118^

## Key interventions in addressing TB among older adults

### TB transmission and infection management

#### Testing for TB infection, disease monitoring, and infection control

Pre-admission screening of risk factors for TB could facilitate early TB case finding.^119^ In Hong Kong SAR, systematic screening of new admissions to the aged-care facilities for TB infection using IGRA and for TB disease using chest X-rays, was highly cost-effective at US$19,712 and US$29,951 per quality-adjusted life-year (QALY) gained; compared to TB screening using GeneXpert® MTB/RIF and no screening.^120^ Further to the screening of residents, environmental control measures such as improved ventilation and selective use of ultraviolet germicidal irradiation in aged-care facilities could reduce TB transmission risk.^121^ Given the risk of infection among residents and staff in aged-care facilities, it is critical to generate evidence and establish clear guidelines for TB infection and disease screening, as well as ongoing TB disease monitoring in settings with high rates of TB infection. Minimum infection control criteria require consideration, with regular reporting and review of practices to limit TB disease and transmission risk.

#### TB preventive treatment (TPT)

Compared to isoniazid-monotherapy for 6–9 months, rifamycin-based combination TPT for 3–4 months has similar efficacy, lower risk of liver injury, and better treatment adherence.^122^ Given that the risk of hepatotoxicity associated with isoniazid use in older adults ≥65 years remains a concern,^59^ 4-month rifampicin monotherapy, endorsed by the WHO for HIV-negative individuals,^57^ could provide a safer alternative for older adults.^123^ While there is empirical evidence on TPT administration, tolerability, and a TPT completion rate (isoniazid and rifampin-based) of >80% among older adults in the Republic of Korea,^124^ there remains a paucity of data and specific guidance on TPT use in this population. The risk of adverse events associated with TPT remains a concern (including the shorter regimen).^125^ Therefore, further assessments on approaches to systematically manage TB infection and TPT in this population are necessary.

Older adults who might be eligible (e.g., at-risk groups such as immunocompromised or documented TB contact) should be carefully assessed, tested for TB infection, and carefully monitored for adverse effects if TPT is initiated. Monitoring should extend beyond TPT administration, and a TPT register should provide an overview of the complete care cascade.^126^ If thorough risk-benefit assessments and emerging evidence recommend TPT to eligible older adults, timely and effective referrals and the integration of TB infection testing and treatment with other health services for chronic diseases should be explored as a pathway to administer TPT effectively.^127^^,^^128^ It is important that the design of relevant policies and programs should be evidence-based and adhere to the clinical standards for managing TB infection.^126^

### TB diagnosis and detection

#### Timely diagnosis

WHO-approved NAATs are recommended as the initial test of choice to diagnose TB given improved sensitivity (compared to smear microscopy) and rapid turnaround time (∼2 h to perform the test) with simultaneous drug resistance prediction.^129^ Therefore, increasing the availability and accessibility of NAATs could facilitate timely diagnosis and treatment initiation.

The incorporation of NAATs in the field, particularly for ACF, was feasible yet resource intensive. Their efficiency hinges on the availability of a comprehensive laboratory and healthcare ecosystem such as workforce capacity, sample transportation, test results communications and follow-up.^130^ A lack thereof, especially in under-resourced settings, could pose a challenge in timely TB diagnosis and treatment.^80^

Interventions should also be implemented to improve the quality of sputum samples collected from older adults, especially those who are frail. More invasive approaches to obtain specimens from the respiratory system and early morning gastric aspirates could be used, but the potential benefit should be weighed against the risks of more invasive procedures.^35^^,^^131^ Among people living with HIV, lateral flow urine lipoarabinomannan assay (LF-LAM) could be considered a complementary tool to assist TB disease diagnosis, especially when sputum collection is impossible or disseminated disease is considered.^132^ LF-LAM's potential value in older adults with immune senescence or other forms of immunocompromise has not been evaluated and requires more formal exploration.

#### Social protection

Social protection in the form of income replacement and financial grants may reduce TB incidence and mortality in older adults.^133^ In Cambodia, the provision of health equity funds to support the poor improved access to healthcare services and reduced the overall medical financial burden.^134^ More specifically, the provision of free TB services, prioritisation of the needs of high-TB risk groups, and reduction of out-of-pocket payments on healthcare under the universal health coverage framework are critical to ensure those affected by the disease have access to the required TB care and rehabilitation.^135^ Interventions are also required to reduce the risk of falling into poverty after a TB diagnosis.^136^ It is noteworthy that progress toward UHC differs by country and area in the region (UHC indices range from 33 in Papua New Guinea to 87 in Australia). Therefore, intensified efforts are required by individual countries to develop a strong and more inclusive health systems for all, including those affected by TB.

#### Active case finding

Interventions to actively seek and diagnose TB disease among older adults are more effective than traditional passive case-finding (PCF).^137^ Targeted screening of specific risk groups (e.g., people with diabetes or a history of TB) using chest radiography as a screening tool helps to detect previously undiagnosed patients among older adults.^138^, ^139^, ^140^ In Cambodia, the use of computer aided detection (CAD) has resulted in an increased yield of TB detection among older adults.^141^ Incorporating novel technology combining ultra-mobile digital chest radiographs and CAD is a promising area for further research and scale-up to improve TB detection. Community-based active case-finding models that target older adults can be effective in settings with variable TB burden.^141^, ^142^, ^143^ The experience in the Republic of Korea and Cambodia demonstrated an increased TB yield among older adults compared to expected case notification rates.^141^^,^^143^^,^^144^ Beyond older adults-specific approaches, community-wide screening for TB using NAAT in high-incidence settings such as Viet Nam was also shown to lower the prevalence and transmission of TB in the population as a whole.^145^ Contact investigation in settings like the Philippines, where TB disease and infection are highly prevalent among TB-exposed household members, demonstrated the value of contact investigation for active case finding and prevention of future disease among individuals and affected families.^146^ However, its impact on community transmission has not been demonstrated. Furthermore, aged-care facilities could consider monitoring and screening healthcare staff and institutionalised older adults for TB disease at regular intervals. Apart from the major benefits of active case finding in high-risk groups, negative aspects related to potential stigma and misdiagnosis should be considered as well.^147^

#### TB and healthcare services organisation and governance

To improve the quality of healthcare for older adults, the WHO has introduced a set of age-friendly healthcare principles that seek to enhance the quality of life for the ageing population.^61^ The adoption of such a framework through training in the core competencies of managing geriatric conditions, enhancing the facility's physical environment, reducing waiting times, introducing an appointment system, and other service improvements should be considered to improve healthcare accessibility for older adults.^61^

The decentralisation of TB services to primary health care was also instrumental in increasing access to health services.^62^ In China, systematic screening of TB disease is done in tandem with the annual health screening offered to older adults at their local hospital free of charge, increasing the efficiency of active TB case-finding.^17^^,^^138^^,^^148^^,^^149^ It has been estimated that the inclusion of active TB case finding during annual health checks in China would result in a 48% and 58% decline in TB disease incidence and mortality, respectively.^17^ Beyond TB case finding, the integration of services also facilitates screening for other diseases and risk factors, particularly co-morbid conditions like undernutrition, cigarette smoking, diabetes, and excessive alcohol use.^150^ Such linkages encourage person-centred care and should improve clinical management for TB and other diseases, with better overall health outcomes.^150^

### TB treatment and adherence

#### Person-centred approaches to treatment monitoring, and support

Person-centred TB care models should include tailored interventions and comprehensive care plans that improve adherence and limit loss to follow-up. These may include customised treatment support that extends beyond directly observed therapy (DOT), as well as education, social and psychological support for people with TB and their close family members.^151^ Facility-based treatment support requires TB patients to visit a health facility daily to administer medications, which can be challenging for older adults.^152^ Use of family or community treatment support has been associated with lower default rates,^153^ higher treatment success rates, and reduced death compared with facility-based treatment support.^154^^,^^155^ Better health services integration would facilitate the monitoring of TB treatment and the management of relevant co-morbidities, improving both the quality and efficiency of care.^156^

While social protection seeks to address the socioeconomic risk factors for TB and facilitates access to health care, a strong social support system is important to assist recognition of TB symptoms, early diagnosis and treatment completion. Studies have shown that social support improves knowledge, attitude, and beliefs regarding TB, positively impacting TB treatment adherence and outcomes.^157^^,^^158^ In China, a comprehensive system that included health education and peer support groups improved the outcomes of older adults treated for TB.^159^

#### Improve digital literacy and access to virtual support

A more person-centred approach could also benefit from the innovative use of new technology. The use of telemedicine, both synchronous (observed live through a video camera) and asynchronous (recorded and sent to treatment observers or health workers for documentation and verification), has been shown to be cost-effective and user-provider friendly in treatment observation.^160^, ^161^, ^162^, ^163^ As smartphone and mobile internet connectivity becomes more accessible than before, the feasibility of technology-enabled solutions increases. This was demonstrated by the COVID-19-induced health crisis, where the adoption of digital innovations allowed TB care to continue with minimal disruptions.^164^^,^^165^ Beyond direct observation and supervision, technological solutions such as electronic medication monitoring devices could be implemented to support and monitor treatment adherence.^166^^,^^167^ However, uptake of these technology-dependent approaches is likely less favourable among older adults.^166^ As technology adoption differs widely across the region, it is vital to understand the contextual determinants of acceptability and access to new technology,^168^ as well as its uptake and utility among older adults.

### Post-TB health and rehabilitation

Recognition of the long-term physical and mental health impacts of TB identifies a need to identify, manage and prevent these ramifications.^116^ Post-TB rehabilitation should be guided by a careful assessment of ongoing physical and psychological needs at the end of TB treatment.

#### Better linkages between health services to assist in monitoring and follow-up

Considering the growing population of people who have survived TB, better linkages between TB, non-communicable diseases, and social and psychological support programs are essential.^169^ If required, post-TB care should include follow-up visits with appropriate healthcare professionals to manage ongoing TB-related complications. Ideally, all older adults should have a regular medical follow-up to assess their general health after TB treatment completion, including the possibility of TB recurrence and appropriate management of all co-morbidities.

#### Data on post-TB outcomes and care needs in older adults

A better understanding of the severity, frequency, and risk factors of adverse post-TB health outcomes^113^^,^^170^ is needed to inform potential interventions. The feasibility of conducting post-TB assessment under routine programmatic conditions has been demonstrated in China, and the experience could be adapted to other settings.^171^

## Future research

Given the rapid growth of the ageing population in the Western Pacific Region and the vulnerability of older adults to TB, more research is required to generate the evidence needed to better prevent and manage TB in this population. Table 2 provides an overview of key knowledge gaps. As aged-care facilities are high-risk environments for TB transmission, the feasibility, effectiveness, and cost-effectiveness of TB screening strategies among institutionalised older adults and their caregivers must be investigated. Beyond aged-care facilities, understanding the access barriers and facilitators of TB service utilisation among older adults is important to assist case finding, treatment adherence and post-treatment follow-up. Understanding all risk factors associated with TB diagnostic delay and poor treatment outcome is essential for improving care. For TB diagnosis, a key challenge is developing an accurate non-sputum-based test. For TB treatment, shorter and safer regimens and more user-friendly approaches to treatment delivery and monitoring are needed. Further down the care cascade, the full range of post-TB sequelae requires better description to enable tailored interventions and care plans for TB survivors. It is also important to consider broader issues such as social protection and relevant ethical principles, with a particular need for more research in low-and-middle-income countries that are also experiencing a demographic transition.

**Table 2:** TB research domains and priorities in older adults.

Domains	Priorities
Transmission and infection management	1. Effectiveness and cost-effectiveness (including risk-benefit analysis) of TB infection testing and TPT for healthcare workers caring for older adults in nursing facilities or healthcare settings
Diagnosis and case-finding	2. Barriers and facilitators in access to and utilisation of health care, particularly TB services 3. Effectiveness and cost-effectiveness of TB screening strategies, including diagnostic tools and frequency, as well as safety of TPT, among institutionalised older adults 4. Quantification of the time to TB diagnosis and treatment initiation 5. Risk factors for delayed care-seeking, diagnosis, and treatment initiation 6. Knowledge, awareness, and practices of healthcare providers on TB screening, detection, and diagnosis among older adults 7. Development of non-sputum-based TB diagnostics 8. Optimal coverage and breadth of social protection services for older adults 9. Ethics, equity, and human rights issues in TB among older adults
Treatment	10. Shorter and safer TB regimens for both TB infection and TB disease for the older adults 11. Effectiveness, cost-effectiveness, and implementation science of innovative approaches, including but not limited to the use of mobile digital technologies and community engagement, in TB treatment administration (e.g., treatment support) and monitoring (adherence, adverse effects, and outcomes) among older adults
Post-TB health and rehabilitation	12. Severity, frequency, risk factors, and costs of post-TB health, including psychological, and social sequelae 13. Effectiveness and cost-effectiveness of practices and interventions to improve post-TB health and wellbeing 14. Palliative and end-of-life care for older adults where TB cure is not feasible

TB; tuberculosis, TPT; TB preventive treatment.

## Conclusion

The TB burden is generally reduced in populations with increased life expectancy, mainly through improved socioeconomic conditions. However, the risk of TB disease in these settings may be increased among older adults with past infection, given that age-associated immune dysfunction increases the risk of TB reactivation. Older adults exhibit atypical features of TB and often present with multiple comorbidities, which may delay TB diagnosis, while their care-seeking behaviour may be influenced by reduced mobility and more difficult interaction with the health care system. Existing comorbidities also complicate TB treatment, due to the higher risk of adverse drug reactions and drug–drug interactions. Post-TB sequelae and ongoing socioeconomic hardship may decrease the quality of life after TB treatment completion.

Age-friendly healthcare infrastructure and services, increased awareness of atypical TB manifestations, and integration of TB case-finding strategies with comorbidity management may assist earlier case detection. Treatment adherence and adverse event monitoring requires increased vigilance and careful consideration of age-considerate technology. Effective infection control measures and routine screening may reduce TB transmission risk, particularly in aged care facilities or other congregate settings.

Further research and innovation, as well as current best practices, should inform the formulation of policies and programmatic guidance to improve TB prevention, detection, and care practices among older adults. Considering the Western Pacific Region's rapidly ageing population and the increasing TB burden observed among older adults, it seems pertinent and timely to develop a roadmap for TB care focussed on this population.

## Contributors

T.I., B.J.M., K.R., F.M., K.H.O., K.V., C.W.M.O., S.K., H.K., Y.L., S.Y., H.T.G.T. conceptualised and designed the study. A.K.J.T. conducted the literature search. B.J.M., C.W.M.O., L.K., F.M., K.V., K.R., M.Y. provided additional references. A.K.J.T. and F.M. drafted the manuscript. B.J.M., K.V., K.H.O., T.I., K.R., S.Y., C.W.M.O., S.K., T.Y., A.O., L.K., H.J.K., Y.L., M.Y., K.P., H.T.G.T. critically revised the manuscript. All authors contributed to the final version of the manuscript, reviewed, and approved the manuscript.

## Data sharing statement

All data included in this paper are available from the reference list.

## Declaration of interests

The authors declare that they have no competing interests.
