# Supplementary material for: Tuberculosis in older adults: challenges and best practices in the Western Pacific Region
Source: Lancet Reg Health West Pac. 2023 Apr 20;36:100770. doi: 10.1016/j.lanwpc.2023.100770 (PMC10398605; doi:10.1016/j.lanwpc.2023.100770)
Supplement: Supplementary material [file mmc1.docx]

**Supplementary material**

Table of Contents

[Methods 1](#_Toc128516029)

[Search strategy and selection criteria 1](#_Toc128516030)

[Limitations 1](#_Toc128516031)

[Supplementary Figure 1: Flow diagram of literature search addressing key practices and challenges in addressing TB among older adults in the Western Pacific Region 2](#_Toc128516032)

[Supplementary Table 1: Summary of key challenges and interventions identified in the peer-reviewed literature to address TB among older adults in the Western Pacific Region, categorised by reported TB incidence rate 3](#_Toc128516033)

[References 7](#_Toc128516034)

# **Methods**

Prior to the commencement of the project, a meeting with all the stakeholders from the region and the World Health Organization (WHO) was held to determine the domains of interest where the following topics, including the application of the TB care cascade framework, were considered⎯1) risk factors for TB among older adults; 2) transmission and reactivation of TB infection; 3) clinical manifestations of TB disease among older adults, and 4) challenges and best practices in managing TB across the full spectrum of infection and disease, including: prevention, detection and diagnosis, treatment and adherence, and post-TB health and wellbeing.

## Search strategy and selection criteria

We performed a narrative review of the available literature on PubMed published between 1996 and 2021. Search terms included a combination of “aging”, “ageing”, “older people”, “elderly”, and “tuberculosis” (free-text index terms and Medical Subject Headings terms), and there were no language restrictions. Boolean operators “OR” and “AND” were used to search for articles relevant to the concept of TB in older adults.

We focused our search on country experiences reported from the Western Pacific Region, including studies that described risk factors for TB disease and transmission, as well as clinical manifestations of TB disease among older adults. Due to the breadth of the topic areas covered, we needed to purposively restrict ourselves to relevant articles from the region, as well as articles providing a more general overview (e.g., systematic reviews, meta-analyses, and narrative reviews). In addition, we reviewed the reference lists of selected articles and consulted a range of regional stakeholders through the WHO Office in Manilla to ensure that key areas within each domain were presented and discussed. We also reviewed the reference list of key articles that reported similar topics previously to identify all relevant studies.

For country-specific experiences, we limited the search to countries and areas in the Western Pacific Region, as defined by WHO.^1^ We included studies that described challenges, interventions, and policies in managing TB among older adults along the care cascade. Works outside the region and among populations not identified as older adults were excluded. In total, we identified 759 unduplicated records, of which 91 full texts were reviewed after initial title screening (**Supplementary** **Figure 1**). Stakeholders and co-authors reviewed multiple iterations of the narrative review and the accompanying reference list. Additional studies/reports were nominated by stakeholders and included where these were considered relevant by the author group.

Extracted data were organised according to the country of study, as well as specific challenges and policies to address TB among older adults. We also presented indicators on country income groups, universal health coverage indices, TB incidence rates and the proportion of incident cases among older adults, as well as Bacillus Calmette–Guérin (BCG) vaccination coverage by countries and areas using data from the World Bank, the United Nations, and World Health Organization.^2–5^ Database searches, articles screening based on the inclusion and exclusion criteria, and information extraction were primarily conducted by the first author (AKJT), and the processes were verified by FM.

## Limitations

The review covers a very broad topic area, encompassing the whole TB care cascade in older adults. This condition does not allow every step to be covered in great depth, but the intent was to provide a useful overview of important themes and challenges, strategies to address these challenges, and consideration of key knowledge and implementation gaps to guide future framework development. Given the region’s diversity, careful contextualisation of our findings is required in different settings. Furthermore, the review was limited by the lack of a systematic selection of articles, evaluation of biases, and it did not consider the individual study sample sizes or the magnitude of effect sizes. Most studies that described country-specific experiences were undertaken in Japan, the Republic of Korea, China, Hong Kong SAR, Singapore, and Cambodia. Therefore, more research is needed in low-and-middle-income countries in the region. The inclusion of grey literature and non-peer-reviewed articles was limited, which may have further narrowed the coverage of information from countries and areas with limited research and peer-reviewed publication outputs.

## **Supplementary Figure 1: Flow diagram of literature search focused on key practices and challenges in addressing TB among older adults in the Western Pacific Region**


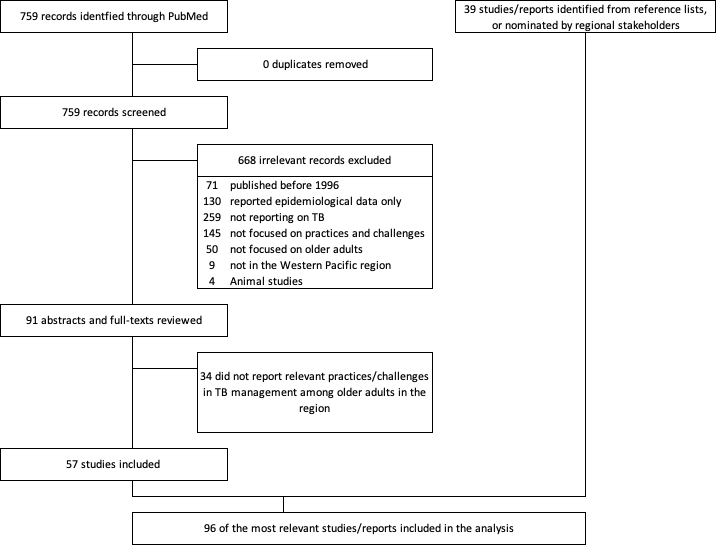


# **Supplementary Table 1: Summary of key challenges and interventions identified in the peer-reviewed literature to address TB among older adults in the Western Pacific Region, categorised by WHO estimated TB incidence rate**

|  | **TB incidence rate (no. of TB cases per 100000 population per year) in 2021^#^** | | |
| --- | --- | --- | --- |
|  | **<50** | **50 – 99** | **≥100** |
| Countries and territories^*^ | Niue, Singapore, Republic of Korea, Guam, Vanuatu, Tokelau, French Polynesia, Cook Islands, Japan, New Caledonia, Tonga^†^, New Zealand^†^, Samoa^†^, Australia^†^, American Samoa^†^, Wallis and Futuna^†^ | Malaysia, Northern Mariana Islands, Micronesia Federal States of, Fiji, Solomon Islands, Brunei Darussalam, Macao SAR, Hong Kong SAR, China^‡^, Palau | Philippines^**^, Marshall Islands^††^, Papua New Guinea^††^, Mongolia^††^, Kiribati^††^, Tuvalu, Cambodia, Nauru, Viet Nam, Lao People's Democratic Republic |
| **Key challenges in addressing TB among older adults^§^** | | | |
| TB transmission and infection management | Japan and Australia: Higher risk of TB infection and disease among older adults residing in aged-care facilities and healthcare workers caring for them^6–11^ | Hong Kong SAR: Higher risk of TB infection and disease among older adults residing in aged-care facilities and healthcare workers caring for them^12^ |  |
|  | Republic of Korea: Loss to follow-up during TB infection treatment^13^ | China: Higher risk of TB infection and disease among older adults residing in aged-care facilities and healthcare workers caring for them^14^ |  |
|  | Age-related immunosenescence and reduction in the sensitivities of TB infection tests^15–20‖^ | | |
|  | Adverse effects of TB preventive therapy regimen^21–24‖^ | | |
| TB diagnosis and detection - Delayed TB care-seeking |  | China: Long waiting time and poor attitude of healthcare staff towards older adults^25^ | Cambodia: Healthcare seeking at facilities that do not provide TB services^26^ |
|  |  | China: Inadequate TB awareness and knowledge among older adults^27,28^ | Cambodia: Lack of knowledge about TB services, its costs, and misconception of TB risks^29^ |
|  |  | China: Fear of discrimination^30^ |  |
|  |  | China: Lack of financial resources^28,30^ |  |
| TB diagnosis and detection - Delayed diagnosis | Japan: Missed diagnosis due to atypical presentations and low awareness among health practitioners in low incidence settings^10,31–33^ |  | Lao PDR: Limited TB diagnostic capacities and rely predominantly on the presentation of TB symptoms^34^ |
|  | Republic of Korea: Missed diagnosis due to atypical presentations^35,36^ |  |  |
|  | TB diagnosis complicated by challenges related to advanced age, such as cognitive impairment, dementia, hearing loss, and speech impairment^37^ | | |
|  | Older adults may struggle to produce adequate expectorated sputum, thereby reducing diagnostic sensitivities^38–40‖^ | | |
|  | Challenges in implementing efficient and sensitive TB diagnostic modalities widely, especially in under-resourced settings^40^ | | |
| TB treatment and adherence | Drug-drug interactions due to polypharmacy for the treatment of existing comorbidities^41,42‖^ | | |
|  | Higher risk of TB drug toxicities, including concomitant use of traditional, complementary, and herbal medicines^43–49‖^ | | |
|  | Republic of Korea: Poor treatment adherence due to comorbidities (e.g., dementia)^50^ and loss to follow-up^51^ | Hong Kong SAR: Poor treatment outcomes among older adults due to comorbidities, socioeconomic disadvantage, and generally more severe disease^52^ |  |
|  |  | China: Poor treatment adherence due to comorbidities and adverse drug reactions; limited understanding of disease process and need for effective treatment^53^ |  |
| Post-TB health and rehabilitation | Higher risk of TB-related chronic lung disease, death, negative impact on quality of life, income, mental health, also of TB and post-TB stigma^54–60‖^ | | |
| **Key interventions in addressing TB among older adults^§^** | | | |
| TB transmission and infection management | Japan: Screening of risk factors for TB before admission to aged-care facilities^61^ | Hong Kong SAR: TB infection and disease screening of new admissions to aged-care facilities using IGRA and chest x-rays^62^ |  |
|  | Japan: TB screening for residents of aged-care facilities and staff caring for them^63^ | China: Integration of TB infection testing and treatment services with other healthcare services^64^ and active case finding activities^65^ |  |
|  | Japan: Implementation of environmental control measures such as improved ventilation and the use of ultraviolet germicidal irradiation^66^ |  |  |
|  | Singapore: Integration of TB infection testing and treatment services with other healthcare services^67^ |  |  |
|  | Integration of TB infection testing and treatment with other health services for chronic diseases as a pathway to effectively administer TPT (if thorough risk-benefit assessments and emerging evidence recommend TPT to eligible older adults)^68^ | | |
| TB diagnosis and detection | Increase nucleic acid amplification test (NAAT) access and the required resources and ecosystem to fully implement NAAT for rapid turnaround time^69,70‖^ | | |
|  | Consider approaches such as urine LF-LAM for older adults living with HIV and other options for TB specimen collection and diagnosis - as appropriate^71–74‖^ | | |
|  | Introduction of age-friendly healthcare services^75‖^ | | |
|  | Social protection and universal health coverage^76,77‖^ | | |
|  | Active case finding:   - Japan: Chest x-ray screening programs prioritizing community dwellers ≥ 80 years at primary care clinics or mass screening events in the community^78^ - Republic of Korea: Systematic screening of older adults using chest x-rays and presence of TB symptoms^79,80^ | Active case finding:   - Malaysia: TB screening among residents of aged-care facilities^81^ - China: TB case finding during annual physical check-up/health examination activities using chest x-rays for older adults with symptoms or risk factors for TB^65,82–84^ - China: Door-to-door household visits and community-based interventions to screen for TB using chest x-rays and TB symptoms among older adults^85–88^ | Active case finding:   - Cambodia: Community-based initiatives using mobile chest x-rays and GeneXpert MTB/RIF^89^ |
|  |  |  | Philippines and Cambodia: Decentralization of TB services to primary health care^90^ |
|  |  |  | Cambodia: Provision of funds to support the poor affected by TB^91^ |
|  | Singapore: Expand DOT to healthcare facilities managing other health conditions. So older adults could receive all their treatments in one place^67^ | China: Social support, health education, and peer support groups^92^ | Viet Nam: Incorporation of technology such as telemedicine to improve treatment monitoring and adherence^93^ |
|  | Japan: Home visit DOT for older adults^94^ | China: Use of non-facility based DOT such as family and community,^95^ and other innovation such as electronic medication monitor as appropriate^96,97^ | Mongolia: Use of community DOT to improve treatment success and reduce death^98^ |
|  | Social support to improve knowledge, attitude, and beliefs regarding TB, which positively impacts TB treatment adherence and outcomes^99,100^ | | |
| Post-TB health and rehabilitation |  | China: Assess post-TB health under routine programmatic settings^101^ |  |

DOT; directly observed therapy, IGRA; interferon-gamma release assay, LF-LAM; lateral flow urine lipoarabinomannan assay, MTB/RIF; Mycobacterium tuberculosis and resistance to rifampin, SAR; Special Administrative Region, TB; tuberculosis, WHO; World Health Organisation

^*^Countries and areas were grouped by TB incidence rates in 2021 (ordered from high to low in the respective groups). No data is available for Pitcairn Island

^†^Countries and areas with TB incidence rates <10 per 100000 population per year in 2021

^‡^China is classified a high TB burden country based on their estimated absolute number of incident cases in 2019 (overall incidence rate in 2021: 55 per 100000 population per year)

^§^Specific challenges and best practices represent country-specific experiences and do not collectively represent all countries and regions in the TB incidence rate groups

^‖^Cross-cutting themes that are not specific to a specific country, area, or TB incidence rate group

^#^Years of evidence generated and TB incidence rates per 1000000 population categorisation (≥500; severely endemic, 300 – 499; highly endemic, 100 – 299; endemic, 50 – 99; upper moderate, 10 – 49; lower moderate, and <10; low incidence) might not match

^††^Countries and areas with TB incidence rates between 300 and 499 per 100000 population per year in 2021

^**^Countries and areas with TB incidence rates ≥500 per 100000 population per year in 2021

# **References**

1 World Health Organization Regional Office for the Western Pacific. World Health Organization: Western Pacific - where we work. 2009. https://www.who.int/westernpacific/about/where-we-work (accessed April 17, 2022).

2 World Bank. World Bank country and lending groups. 2022. https://datahelpdesk.worldbank.org/knowledgebase/articles/906519 (accessed July 23, 2022).

3 Sachs J, Lafortune G, Kroll C, Fuller G, Woelm F. Sustainable Development Report 2022. From crisis to sustainable developments: the SDGs as roadmap to 2030 and beyond. Cambridge: Cambridge University Press, 2022 https://dashboards.sdgindex.org/ (accessed July 23, 2022).

4 World Health Organization. Global tuberculosis report 2021. Geneva: World Health Organization, 2021.

5 World Health Organization. Bacillus Calmette–Guérin (BCG) vaccination coverage. 2022. https://immunizationdata.who.int/pages/coverage/BCG.html?YEAR=&CODE= (accessed July 23, 2022).

6 Seto J, Wada T, Suzuki Y, *et al.* Mycobacterium tuberculosis Transmission among Elderly Persons, Yamagata Prefecture, Japan, 2009–2015. *Emerg Infect Dis* 2017; **23**: 448–55.

7 Kondo A, Oketani N, Kuwabara K, *et al.* [An outbreak of pulmonary tuberculosis probably due to exogenous reinfection at a nursing home for the elderly]. *Kekkaku* 2002; **77**: 401–8.

8 Yanagihara H. [Risk of tuberculosis infection among care workers during an outbreak of tuberculosis at a care facility for the elderly]. *Kekkaku* 2014; **89**: 631–6.

9 Moyo N, Trauer J, Trevan P, *et al.* Tuberculosis screening in an aged care residential facility in a low-incidence setting. *Commun Dis Intell Q Rep* 2017; **41**: E209–11.

10 Suzuki N. Preventing tuberculosis infection in healthcare settings. *Kekkaku* 2019; **94**: 569–73.

11 Suzuki Y, Sone T. [A study on preventive measures against tuberculosis in care facilities for the elderly in a Tokyo metropolitan district]. *Kekkaku* 2011; **86**: 437–44.

12 Leung EC, Leung CC, Chang KC, *et al.* Delayed diagnosis of tuberculosis: risk factors and effect on mortality among older adults in Hong Kong. *Hong Kong Med J* 2018; **24**: 361–8.

13 Noh CS, Kim HI, Choi H, *et al.* Completion rate of latent tuberculosis infection treatment in patients aged 65 years and older. *Respir Med* 2019; **157**: 52–8.

14 Lin S-Y, Chien J-Y, Chiang H-T, *et al.* Ambulatory independence is associated with higher incidence of latent tuberculosis infection in long-term care facilities in Taiwan. *J Microbiol Immunol Infect* 2021; **54**: 319–26.

15 Cho K, Cho E, Kwon S, *et al.* Factors Associated with Indeterminate and False Negative Results of QuantiFERON-TB Gold In-Tube Test in Active Tuberculosis. *Tuberc Respir Dis (Seoul)* 2012; **72**: 416–25.

16 Hang NTL, Lien LT, Kobayashi N, *et al.* Analysis of factors lowering sensitivity of interferon-γ release assay for tuberculosis. *PLoS One* 2011; **6**: e23806.

17 Kamiya H, Ikushima S, Kondo K, *et al.* Diagnostic performance of interferon-gamma release assays in elderly populations in comparison with younger populations. *J Infect Chemother* 2013; **19**: 217–22.

18 Kwon Y-S, Kim YH, Jeon K, *et al.* Factors that Predict Negative Results of QuantiFERON-TB Gold In-Tube Test in Patients with Culture-Confirmed Tuberculosis: A Multicenter Retrospective Cohort Study. *PLoS One* 2015; **10**: e0129792.

19 Chan-Yeung M, Dai DLK, Cheung AHK, *et al.* Tuberculin skin test reaction and body mass index in old age home residents in Hong Kong. *J Am Geriatr Soc* 2007; **55**: 1592–7.

20 Eom JS, Kim I, Kim W-Y, *et al.* Household tuberculosis contact investigation in a tuberculosis-prevalent country. *Medicine (Baltimore)* 2018; **97**: e9681.

21 Chong KC, Leung CC, Yew WW, *et al.* Mathematical modelling of the impact of treating latent tuberculosis infection in the elderly in a city with intermediate tuberculosis burden. *Sci Rep* 2019; **9**: 4869.

22 Sterling TR, Villarino ME, Borisov AS, *et al.* Three months of rifapentine and isoniazid for latent tuberculosis infection. *N Engl J Med* 2011; **365**: 2155–66.

23 Smith BM, Schwartzman K, Bartlett G, Menzies D. Adverse events associated with treatment of latent tuberculosis in the general population. *CMAJ* 2011; **183**: E173–9.

24 Gao L, Zhang H, Xin H, *et al.* Short-course regimens of rifapentine plus isoniazid to treat latent tuberculosis infection in older Chinese patients: a randomised controlled study. *Eur Respir J* 2018; **52**: 1801470.

25 Hamiduzzaman M, De Bellis A, Abigail W, Kalaitzidis E. The Social Determinants of Healthcare Access for Rural Elderly Women - A Systematic Review of Quantitative Studies. *The Open Public Health Journal* 2017; **10**. DOI:10.2174/1874944501710010244.

26 Teo AKJ, Ork C, Eng S, *et al.* Determinants of delayed diagnosis and treatment of tuberculosis in Cambodia: a mixed-methods study. *Infectious Diseases of Poverty* 2020; **9**: 49.

27 Wang Y, Gan Y, Zhang J, *et al.* Analysis of the current status and associated factors of tuberculosis knowledge, attitudes, and practices among elderly people in Shenzhen: a cross-sectional study. *BMC Public Health* 2021; **21**: 1163.

28 Yan F, Thomson R, Tang S, *et al.* Multiple perspectives on diagnosis delay for tuberculosis from key stakeholders in poor rural China: Case study in four provinces. *Health Policy* 2007; **82**: 186–99.

29 Yi S, Teo AKJ, Sok S, *et al.* Barriers in access to services and information gaps by genders and key populations in the national Tuberculosis programme in Cambodia. *Global Public Health* 2021; : 1–14.

30 Wang Y, Feng J, Zhang J, *et al.* Willingness to seek medical care for tuberculosis and associated factors among the elderly population in Shenzhen: a cross-sectional study. *BMJ Open* 2021; **11**: e051291.

31 Nakao M, Sone K, Kagawa Y, *et al.* Diagnostic delay of pulmonary tuberculosis in patients with acute respiratory distress syndrome associated with aspiration pneumonia: Two case reports and a mini-review from Japan. *Exp Ther Med* 2016; **12**: 835–9.

32 Hikone M, Ainoda Y, Sakamoto N, Ohnishi K. Clinical characteristics of elderly pulmonary tuberculosis in an acute-care general hospital in Tokyo, Japan: A 12-year retrospective study. *Journal of Infection and Chemotherapy: Official Journal of the Japan Society of Chemotherapy* 2020; **26**: 245–50.

33 Toyota E, Machida K, Nagayama N, *et al.* [Clinical investigation among elderly patients with tuberculosis]. *Kekkaku* 2010; **85**: 655–60.

34 Law I, Sylavanh P, Bounmala S, *et al.* The first national tuberculosis prevalence survey of Lao PDR (2010-2011). *Tropical medicine & international health: TM & IH* 2015; **20**: 1146–54.

35 Kim HW, Myong J-P, Kim JS. Estimating the burden of nosocomial exposure to tuberculosis in South Korea, a nationwide population based cross-sectional study. *Korean J Intern Med* 2021; **36**: 1134–45.

36 Heo D-H, Seo J-W, Kim J-H, *et al.* Delays in Isolating Patients Admitted to Hospital with Pulmonary Tuberculosis in Korea. *Journal of Korean Medical Science* 2019; **34**: e270.

37 Sharma M, Onozaki I, Nunn P. TB in older people in Asia: why it is important. *The International Journal of Tuberculosis and Lung Disease* 2021; **25**: 521–4.

38 Negin J, Abimbola S, Marais BJ. Tuberculosis among older adults--time to take notice. *Int J Infect Dis* 2015; **32**: 135–7.

39 Rieder HL, Lauritsen JM, Naranbat N, Katamba A, Laticevschi D, Mabaera B. Quantitative differences in sputum smear microscopy results for acid-fast bacilli by age and sex in four countries. *Int J Tuberc Lung Dis* 2009; **13**: 1393–8.

40 Parsons LM, Somoskövi Á, Gutierrez C, *et al.* Laboratory Diagnosis of Tuberculosis in Resource-Poor Countries: Challenges and Opportunities. *Clin Microbiol Rev* 2011; **24**: 314–50.

41 Salazar JA, Poon I, Nair M. Clinical consequences of polypharmacy in elderly: expect the unexpected, think the unthinkable. *Expert Opinion on Drug Safety* 2007; **6**: 695–704.

42 Riccardi N, Canetti D, Rodari P, *et al.* Tuberculosis and pharmacological interactions: A narrative review. *Current Research in Pharmacology and Drug Discovery* 2021; **2**: 100007.

43 Kwon BS, Kim Y, Lee SH, *et al.* The high incidence of severe adverse events due to pyrazinamide in elderly patients with tuberculosis. *PLoS One* 2020; **15**: e0236109.

44 Hase I, Toren KG, Hirano H, *et al.* Pulmonary Tuberculosis in Older Adults: Increased Mortality Related to Tuberculosis Within Two Months of Treatment Initiation. *Drugs Aging* 2021; **38**: 807–15.

45 Devarbhavi H, Aithal G, Treeprasertsuk S, *et al.* Drug-induced liver injury: Asia Pacific Association of Study of Liver consensus guidelines. *Hepatol Int* 2021; **15**: 258–82.

46 Takamatsu A, Kano Y, Tagashira Y, Kirikae T, Honda H. Current in-hospital management for patients with tuberculosis in a high-income country: a retrospective cohort study. *Clinical Microbiology and Infection* 2021; **0**. DOI:10.1016/j.cmi.2021.07.011.

47 Li X, Li X, Liu Q, *et al.* Traditional Chinese medicine combined with western medicine for the treatment of secondary pulmonary tuberculosis: A PRISMA-compliant meta-analysis. *Medicine (Baltimore)* 2020; **99**: e19567.

48 Liu Q, Garner P, Wang Y, Huang B, Smith H. Drugs and herbs given to prevent hepatotoxicity of tuberculosis therapy: systematic review of ingredients and evaluation studies. *BMC Public Health* 2008; **8**: 365.

49 Wada M. [Effectiveness and problems of PZA-containing 6-month regimen for the treatment of new pulmonary tuberculosis patients]. *Kekkaku* 2001; **76**: 33–43.

50 Bea S, Lee H, Kim JH, *et al.* Adherence and Associated Factors of Treatment Regimen in Drug-Susceptible Tuberculosis Patients. *Front Pharmacol* 2021; **12**: 625078.

51 Son H, Mok J, Lee M, *et al.* Status and Determinants of Treatment Outcomes Among New Tuberculosis Patients in South Korea: A Retrospective Cohort Study. *Asia Pac J Public Health* 2021; **33**: 907–13.

52 Leung CC, Yew WW, Chan CK, *et al.* Tuberculosis in older people: a retrospective and comparative study from Hong Kong. *Journal of the American Geriatrics Society* 2002; **50**: 1219–26.

53 Bele S, Jiang W, Lu H, *et al.* Population aging and migrant workers: bottlenecks in tuberculosis control in rural China. *PLoS One* 2014; **9**: e88290.

54 Fox GJ, Nguyen VN, Dinh NS, *et al.* Post-treatment Mortality Among Patients With Tuberculosis: A Prospective Cohort Study of 10 964 Patients in Vietnam. *Clin Infect Dis* 2019; **68**: 1359–66.

55 Marais BJ, Chakaya J, Swaminathan S, *et al.* Tackling long-term morbidity and mortality after successful tuberculosis treatment. *The Lancet Infectious Diseases* 2020; **20**: 641–2.

56 Pasipanodya JG, Miller TL, Vecino M, *et al.* Using the St. George respiratory questionnaire to ascertain health quality in persons with treated pulmonary tuberculosis. *Chest* 2007; **132**: 1591–8.

57 Basham CA, Romanowski K, Johnston JC. Life after tuberculosis: planning for health. *The Lancet Respiratory Medicine* 2019; **7**: 1004–6.

58 Meghji J, Gregorius S, Madan J, *et al.* The long term effect of pulmonary tuberculosis on income and employment in a low income, urban setting. *Thorax* 2021; **76**: 387–95.

59 Rhee CK, Yoo KH, Lee JH, *et al.* Clinical characteristics of patients with tuberculosis-destroyed lung. *Int J Tuberc Lung Dis* 2013; **17**: 67–75.

60 Park HJ, Byun MK, Kim HJ, *et al.* History of pulmonary tuberculosis affects the severity and clinical outcomes of COPD. *Respirology* 2018; **23**: 100–6.

61 Ohmori M, Wada M, Mitarai S, *et al.* [Tuberculosis control in health care facilities for the elderly, from the viewpoint of risk management]. *Kekkaku* 2006; **81**: 71–7.

62 Li J, Yip BHK, Leung C, *et al.* Screening for latent and active tuberculosis infection in the elderly at admission to residential care homes: A cost-effectiveness analysis in an intermediate disease burden area. *PLoS ONE* 2018; **13**. DOI:10.1371/journal.pone.0189531.

63 Uchimura K, Ngamvithayapong-Yanai J, Kawatsu L, *et al.* Characteristics and treatment outcomes of tuberculosis cases by risk groups, Japan, 2007–2010. *Western Pacific Surveillance and Response Journal : WPSAR* 2013; **4**: 11–8.

64 Huang H-L, Huang W-C, Lin K-D, *et al.* Completion Rate and Safety of Programmatic Screening and Treatment for Latent Tuberculosis Infection in Elderly Patients With Poorly Controlled Diabetic Mellitus: A Prospective Multicenter Study. *Clin Infect Dis* 2021; **73**: e1252–60.

65 Huynh GH, Klein DJ, Chin DP, *et al.* Tuberculosis control strategies to reach the 2035 global targets in China: the role of changing demographics and reactivation disease. *BMC Med* 2015; **13**: 88.

66 Furuya H. Estimation of environmental control measures for tuberculosis transmission in care facilities for the elderly. *Tokai J Exp Clin Med* 2013; **38**: 135–41.

67 Tam G, Lai SW. Is Singapore on track to eliminate tuberculosis by 2030? A policy case study. *SAGE Open Medicine* 2019; **7**. DOI:10.1177/2050312119851331.

68 Huang H-L, Huang W-C, Lin K-D, *et al.* Completion Rate and Safety of Programmatic Screening and Treatment for Latent Tuberculosis Infection in Elderly Patients With Poorly Controlled Diabetic Mellitus: A Prospective Multicenter Study. *Clinical Infectious Diseases: An Official Publication of the Infectious Diseases Society of America* 2021; **73**: e1252–60.

69 Camelique O, Scholtissen S, Dousset J-P, Bonnet M, Bastard M, Hewison C. Mobile community-based active case-finding for tuberculosis among older populations in rural Cambodia. *Int J Tuberc Lung Dis* 2019; **23**: 1107–14.

70 World Health Organization. WHO consolidated guidelines on tuberculosos. Module 3: diagnosis - rapid diagnostics for tuberculosis detection. 2021 update. Geneva, 2021 https://www.who.int/publications/i/item/9789240029415.

71 Rajagopalan S. Tuberculosis and Aging: A Global Health Problem. *CLIN INFECT DIS* 2001; **33**: 1034–9.

72 Pérez-Guzmán C, Vargas MH, Torres-Cruz A, Villarreal-Velarde H. Does Aging Modify Pulmonary Tuberculosis?: A Meta-Analytical Review. *Chest* 1999; **116**: 961–7.

73 Bjerrum S, Schiller I, Dendukuri N, *et al.* Lateral flow urine lipoarabinomannan assay for detecting active tuberculosis in people living with HIV. *The Cochrane Database of Systematic Reviews* 2019; **2019**. DOI:10.1002/14651858.CD011420.pub3.

74 Konno A, Narumoto O, Matsui H, *et al.* The benefit of stool mycobacterial examination to diagnose pulmonary tuberculosis for adult and elderly patients. *Journal of Clinical Tuberculosis and Other Mycobacterial Diseases* 2019; **16**: 100106.

75 World Health Organization. Active ageing: towards age-friendly primary health care. Geneva: World Health Organization, 2004 https://apps.who.int/iris/bitstream/handle/10665/43030/9241592184.pdf?sequence=1&isAllowed=y (accessed Oct 14, 2021).

76 Carter DJ, Glaziou P, Lönnroth K, *et al.* The impact of social protection and poverty elimination on global tuberculosis incidence: a statistical modelling analysis of Sustainable Development Goal 1. *The Lancet Global Health* 2018; **6**: e514–22.

77 Ban K. Building a tuberculosis-free world on a foundation of universal health coverage. *Lancet* 2019; **393**: 1268–70.

78 Kato S. TB in elderly: experience in Japan. 2022; published online April.

79 Kim H, Kim H-J, Oh K-H, Oh H-W, Choi H. A Pilot Project of Systematic Tuberculosis Screening in the Elderly in a South Korean Province. *Tuberc Respir Dis (Seoul)* 2019; **82**: 194–200.

80 Lee SH. Active Case Finding in the Elderly Tuberculosis in South Korea. *Tuberc Respir Dis (Seoul)* 2019; **82**: 261–3.

81 Mohd Hassan NZA, Razali A, Shahari MR, *et al.* Cost-Effectiveness Analysis of High-Risk Groups Tuberculosis Screening in Malaysia. *Front Public Health* 2021; **9**: 699735.

82 Zhang C, Xia L, Rainey JJ, *et al.* Findings from a pilot project to assess the feasibility of active tuberculosis case finding among seniors in rural Sichuan Province, China, 2017. *PLoS One* 2019; **14**: e0214761.

83 Li J, Liu X-Q, Jiang S-W, *et al.* Improving tuberculosis case detection in underdeveloped multi-ethnic regions with high disease burden: a case study of integrated control program in China. *Infect Dis Poverty* 2017; **6**: 151.

84 Zhang X-L, Li S-G, Li H-T, *et al.* Integrating tuberculosis screening into annual health examinations for the rural elderly improves case detection. *Int J Tuberc Lung Dis* 2015; **19**: 787–91.

85 Chen C, Yang C-G, Gao X, *et al.* Community-based active case finding for tuberculosis in rural western China: a cross-sectional study. *Int J Tuberc Lung Dis* 2017; **21**: 1134–9.

86 Cheng J, Sun Y-N, Zhang C-Y, *et al.* Incidence and risk factors of tuberculosis among the elderly population in China: a prospective cohort study. *Infect Dis Poverty* 2020; **9**: 13.

87 Cheng J, Wang L, Zhang H, Xia Y. Diagnostic Value of Symptom Screening for Pulmonary Tuberculosis in China. *PLoS ONE* 2015; **10**: e0127725.

88 Liu K, Peng Y, Zhou Q, *et al.* Assessment of active tuberculosis findings in the eastern area of China: A 3-year sequential screening study. *International journal of infectious diseases: IJID: official publication of the International Society for Infectious Diseases* 2019; **88**: 34–40.

89 Codlin AJ, Monyrath C, Ky M, Gerstel L, Creswell J, Eang MT. Results from a roving, active case finding initiative to improve tuberculosis detection among older people in rural cambodia using the Xpert MTB/RIF assay and chest X-ray. *Journal of Clinical Tuberculosis and Other Mycobacterial Diseases* 2018; **13**: 22–7.

90 Onozaki I, Law I, Sismanidis C, Zignol M, Glaziou P, Floyd K. National tuberculosis prevalence surveys in Asia, 1990–2012: an overview of results and lessons learned. *Tropical Medicine & International Health* 2015; **20**: 1128–45.

91 Jacobs B, Bajracharya A, Saha J, *et al.* Making free public healthcare attractive: optimizing health equity funds in Cambodia. *Int J Equity Health* 2018; **17**. DOI:10.1186/s12939-018-0803-3.

92 Li X, Wang B, Tan D, *et al.* Effectiveness of comprehensive social support interventions among elderly patients with tuberculosis in communities in China: a community-based trial. *J Epidemiol Community Health* 2018; **72**: 369–75.

93 Nguyen TA, Pham MT, Nguyen TL, *et al.* Video Directly Observed Therapy to support adherence with treatment for tuberculosis in Vietnam: A prospective cohort study. *Int J Infect Dis* 2017; **65**: 85–9.

94 Hoshino H, Kobayashi N. [Evaluation of effect of community DOTS on treatment outcomes by TB surveillance data]. *Kekkaku* 2006; **81**: 591–602.

95 Zhang H, Ehiri J, Yang H, Tang S, Li Y. Impact of Community-Based DOT on Tuberculosis Treatment Outcomes: A Systematic Review and Meta-Analysis. *PLOS ONE* 2016; **11**: e0147744.

96 Wang N, Shewade HD, Thekkur P, *et al.* Electronic medication monitor for people with tuberculosis: Implementation experience from thirty counties in China. *PLoS One* 2020; **15**: e0232337.

97 Wang N, Guo L, Shewade HD, *et al.* Effect of using electronic medication monitors on tuberculosis treatment outcomes in China: a longitudinal ecological study. *Infectious Diseases of Poverty* 2021; **10**: 29.

98 Dobler CC, Korver S, Batbayar O, *et al.* Success of community-based directly observed anti-tuberculosis treatment in Mongolia. *Int J Tuberc Lung Dis* 2015; **19**: 657–62.

99 Hoorn R, Jaramillo E, Collins D, Gebhard A, Hof S. The Effects of Psycho-Emotional and Socio-Economic Support for Tuberculosis Patients on Treatment Adherence and Treatment Outcomes – A Systematic Review and Meta-Analysis. *PLOS ONE* 2016; **11**: e0154095.

100 Munro SA, Lewin SA, Smith HJ, Engel ME, Fretheim A, Volmink J. Patient Adherence to Tuberculosis Treatment: A Systematic Review of Qualitative Research. *PLOS Medicine* 2007; **4**: e238.

101 Lin Y, Liu Y, Zhang G, *et al.* Is It Feasible to Conduct Post-Tuberculosis Assessments at the End of Tuberculosis Treatment under Routine Programmatic Conditions in China? *Tropical Medicine and Infectious Disease* 2021; **6**: 164.
